# Dating and relationship violence among 16–19 year olds in England and Wales: a cross-sectional study of victimization

**DOI:** 10.1093/pubmed/fdx139

**Published:** 2017-11-10

**Authors:** Honor Young, Catherine Turney, James White, Chris Bonell, Ruth Lewis, Adam Fletcher

**Affiliations:** 1 DECIPHer, Cardiff University, Cardiff, UK; 2 DECIPHer, Centre for Trials Research, Cardiff University, Cardiff, UK; 3 Department of Social and Environmental Health Research, London School of Hygiene and Tropical Medicine, London, UK; 4 Department of Sociology, University of the Pacific, 3601 Pacific Avenue, Stockton, CA, USA; 5 Y Lab, Cardiff University, Cardiff, UK

**Keywords:** young people, educational settings, violence

## Abstract

**Background:**

Dating and relationship violence (DRV) is under-researched in the UK, especially among Further Education (FE) students. This study examines the association between DRV victimization and socio-demographic characteristics, sexual identity and dating and relationship behaviours among 16–19 year olds FE students.

**Methods:**

Cross-sectional self-report data were collected from 1751 students aged 16–19 at six FE settings in England and Wales. Factor analysis examined the structure of DRV victimization by gender. Multilevel logistic regression examined the odds ratios of DRV victimization according to socio-demographics, sexual identity and dating behaviours.

**Results:**

DRV victimization clusters into two categories for females, and three for males. Among females, 46.1% experienced controlling behaviours and 31.6% threatening behaviours; 49.9% of males experienced controlling behaviours, 27.1% threatening behaviours and 5.8% online sexual violence. The odds of DRV victimization were 2–8 times greater for males and 2–4 times greater for females who had ever sent a sexually explicit image. No consistent association was found between DRV and age, spending money per week, educational attainment or meeting partners online.

**Conclusions:**

The high prevalence, absence of gender differences and social patterning, suggests DRV victimization may be becoming normalized and is of significant public health importance for young people in England and Wales.

Dating and relationship violence (DRV) encompasses threats, emotional abuse, coercion and controlling behaviours, physical violence, and coerced, non-consensual or abusive sexual activities perpetrated by a current or former casual or steady partner.^1,2^ Globally, 10–50% of women report violence from current or previous partners,^3^ with adolescence a particular risk period.^4^ Cross-sectional studies in the USA and UK indicate that DRV victimization is typically higher among young women than men.^5,6^ In England, a recent survey of 14–17 year olds found 66–75% of young women reported DRV victimization, compared to 32–50% of young men.^5,7^ In a cross-sectional UK study of almost 1500 13–16 year olds, 25% of females reported that a partner had pushed, slapped, hit or held them down on one or more occasions, and 11% of females had been punched, strangled beaten up or hit with an object one or more times.^7^

Early DRV victimization is associated with substance misuse, sexually transmitted infections (STIs) and teenage pregnancy^8^ eating disorders, mental health problems, anti-social behaviour^9^ and violence in adulthood.^10^ In 2008, domestic violence was estimated to cost the UK National Health Service (NHS) £1.73 bn per year.^11^

Evidence of associations between socio-demographic factors and adolescent DRV victimization is equivocal, with most studies undertaken in North America. A review of 61 studies reported lower socio-economic status (SES) was associated with an increased risk for DRV victimization.^12^ Other nationally representative population-based studies report an inverse relationship,^13,14^ whereas others found no association.^15^ Similarly, few consistent associations have been found between ethnicity and DRV victimization,^12^ with some North American studies reporting no association,^14,16^ and others, higher rates in ethnic minority groups.^17^ Other non-nationally representative US research has found slightly lower rates^18^ and UK studies, higher rates of DRV victimization for ethnic minority groups.^7^ Similarly, little research has explored DRV among adolescent same-sex partners. The National Longitudinal Study of Adolescent Health reports that adolescents with same-sex partners have rates of DRV that are lower or equal to rates reported by adolescents with opposite-sex partners,^19^ whereas other longitudinal US^20^ and cross-sectional UK studies report higher victimization rates in same-sex compared to heterosexual adolescents.^7^

The prevalence of meeting partners online and sharing of sexually explicit images in young people has received relatively little empirical attention.^21^ A US study identified that 24% of teenagers who had dated, met their romantic partner online,^22^ however, UK evidence is both limited and mixed as to whether meeting partners online is associated with increased risk.^23^ Internationally, rates for sending sexually explicit images vary from 3 to 34%.^21^ In England, 32% of 14–15 year olds males and 44% of females reported sending a sexual image or text message.^5^ Sending sexually explicit images has been associated with early sexual behaviour, multiple sexual partners, non-contraception use^24^ and taking alcohol or drugs before sex (girls only).^25^ A recent study of five European countries identified a significant association between sending sexually explicit images and face-to-face emotional and physical victimization for both boys and girls.^26^ In all countries, boys and girls were around twice as likely to have sent their partner a sexual image or text if they were a victim of emotional partner violence compared to those who were not victimized. Despite young people being the largest users of mobile phone technology and social media^27^ and adolescence being a key stage in the life course where norms of sexual activity are established, young people engage in sexual risk taking and develop independence and autonomy, there have been few studies examining the association between sending sexually explicit images and DRV among 16–19 year olds in the UK.

Although DRV is more widely recognized and researched within the US, it is still largely under-studied in the UK, especially among young people.^28^ A greater understanding of how victimization clusters among young people, the distribution, the prevalence, the associated socio-demographic, contextual and behavioural factors and consequences for health of the population in the UK, especially among young people, is required to inform intervention and policy development.^28,29^ The challenges of measuring DRV have been discussed^9,30^ and the UK Home Office definition reflects a continuum of DRV. To establish a suitable measure of DRV, this article considers the prevalence of different forms of DRV within a relationship, together with the severity and frequency of these behaviours, relative to young people in England and Wales. In this context, less severe behaviours occurring only once may not be considered to constitute DRV whereas other, more serious behaviours happening even once may be sufficient to warrant DRV classification.

In England, the age at which most young people leave education has been raised to 18 years. Further Education (FE) settings are educational settings that primarily serve 16–19 year olds. There are now more than 1.5 million young people aged 16–19 studying in FE, with increasing participation across all social groups. They are environments where young people are socialized into gender norms and where significant amounts of gender-based harassment and DRV go unchallenged.^31^ Although there is strong evidence overall for a comprehensive, ‘health promoting schools’ approach,^32,33^ there is limited evidence on its application for sexual health or in FE settings. Comprehensive sexual health interventions in US high schools show promising results but they have not been developed for use in UK FE settings.^34^ The British FE sector is unique; its rapid expansion and as the only universal setting for delivering comprehensive sexual health interventions at this key period, it offers a key context for developing and delivering interventions to address these public health priorities in this high-risk age group at population-wide scale.

The evidence is mixed as to whether certain socio-demographic characteristics and dating and relationship behaviours are associated with more experience of DRV. This article provides the first comprehensive estimate of the distribution of dating and relationship violence and of risk and variation of DRV according to socio-demographic and behavioural factors with a large sample of FE students in England and Wales. Establishing the association between socio-demographic, contextual and behavioural characteristics with DRV will help to inform whether universal or targeted interventions are appropriate.^35^ The analysis addresses the following research questions:

1. What is the prevalence and clustering of DRV victimization by 16–19 year olds in FE settings?
2. What is the association between DRV victimization and socio-demographic characteristics, sexual identity, and dating and relationship behaviours for 16–19 year olds in FE settings?

## Method

Cross-sectional data were collected from six FE settings across England (*n* = 3) and Wales (*n* = 3) between September and December 2015, as part of a mixed method, multi-case study to inform the development of a sex and relationships intervention for FE settings. Settings were purposively recruited to reflect different institutional contexts within the sector: two ‘sixth form’ colleges attached to schools (England *n* = 1, Wales *n* = 1), and four large FE college campuses (England *n* = 2, Wales *n* = 2) with a yearly intake of >1000 students.

Multiple modes of recruitment were used to invite all students aged 16–19 to participate. Information about the study and a weblink to the electronic (e)-questionnaire were emailed to all students using their institutional email where possible. Students also completed questionnaires during scheduled lesson time using electronic tablets. Trained fieldworkers attended each data collection session. The majority (58%) of questionnaires were completed electronically with others completed via pen and paper copy due to limited Internet/tablet access.

Ethical approval was sought from the Cardiff University School of Social Sciences Research Ethics Committee. Participants were aged 16 or over and, based on college guidance, deemed as having full capacity to provide informed consent.^36^ Students were provided with written descriptions of the study and provided consent prior to participation. Students had the opportunity to withdraw from the data collection session at any time, and were given contact details for organizations providing relevant information and support following completion of the questionnaire.

### Participants

Data were collected from 2105 students aged 16–19. Participant numbers varied by site with fewer students from ‘sixth form’ colleges (England *n* = 70, 3.33%; Wales *n* = 146, 6.94%), than larger FE campuses (England *n* = 534, 25.37%; *n* = 160, 7.60%, Wales *n* = 616, 29.26%, *n* = 579, 27.51%). Of those participating, 83.2% (*n* = 1751) had dated or been in a relationship. These were used in all subsequent analyses. Over half the sample were female (54%). Participants who did not report a gender (*n* = 2), reported gender combinations (*n* = 3) or ‘other’ gender (*n* = 8) were removed from analyses. The sample consisted of mostly White British (87.1%), heterosexual (90.8%) 16–17 year olds (61.1%) (Table 1). Overall, 13% reported Black or Minority Ethnic group (BME) status and only 4% were living independently. Almost a third of students had less than £20 to spend for themselves each week, and a similar proportion reported low educational attainment (<5 General Certificates of Secondary Education (GCSEs) A*–C). Approximately two-thirds of the sample reported studying on a non-academic educational pathway.

**Table 1:** Sample characteristics and prevalence of dating and relationship violence

	Males (*n* = 797) % (*n*)	Missing % (*n*)	Females (*n* = 954) % (*n*)	Missing % (*n*)	Overall (1751) % (*n*)	Missing % (*n*)
Socio-demographic characteristics						
Percent (*n*) aged 16–17	61.1 (487)	–	67.5 (644)	–	64.6 (1131)	–
Percent (*n*) BME,	12.9 (103)	0.1 (1)	10.9 (104)	0.3 (3)	11.8 (207)	0.2 (4)
Living independently, % (*n*)	3.5 (28)	0.4 (3)	3.5 (33)	1.4 (13)	3.5 (61)	0.9 (16)
£20 or under spending money per week	29.6 (236)	4.4 (35)	36.2 (345)	10.2 (97)	33.1 (581)	7.5 (132)
Low educational attainment <5 GCSEs	30.1 (240)	0.5 (4)	23.6 (225)	1.4 (13)	26.6 (465)	1.0 (17)
Non-academic educational pathway	67.5 (538)	0.8 (6)	56.9 (543)	1.3 (12)	61.7 (1081)	1.0 (18)
Sexual identity						
Heterosexual	90.8 (724)		86.9 (829)		88.7 (1553)	
Homosexual	2.4 (19)	0.5 (4)	2.31 (22)	0.6 (6)	2.3 (41)	0.6 (10)
Bisexual	4.1 (33)		7.4 (71)		5.9 (104)	
Rather not say	1.5 (12)		1.5 (14)		1.5 (26)	
Other	0.6 (5)		1.3 (12)		1.0 (17)	
Dating behaviours						
Ever had a ‘boyfriend/girlfriend’	82.3 (797)	3.2 (31)	83.9 (954)	2.1 (27)	83.2 (1751)	2.8 (58)
Experience of meeting partners online	11.5 (92)	1.9 (15)	13.8 (132)	1.6 (15)	12.8 (224)	1.7 (30)
Sent sexually explicit image	44.5 (355)	4.5 (36)	46.3 (442)	2.3 (22)	45.5 (797)	3.3 (58)
Dating and relationship violence items						
Have any of your boyfriend(s) or girlfriend(s), or anyone you have been ‘seeing’ or ‘dating’ ever?						
Told you who you could see or where you could go						
Never	68.9 (549)	2.3 (18)	66.5 (634)	1.6 (15)	67.6 (1183)	1.9 (33)
Once	10.5 (84)		10.3 (98)		10.4 (182)	
Few times	13.2 (105)		16.6 (158)		15.0 (263)	
Often	5.1 (41)		5.1 (49)		5.1 (90)	
Constantly checked up on what you were doing (e.g. by phone or texts)						
Never	44.9 (358)	2.4 (19)	49.3 (470)	1.4 (13)	47.3 (828)	1.8 (32)
Once	10.9 (87)		10.4 (99)		10.6 (186)	
Few times	29.2 (233)		25.7 (245)		27.3 (478)	
Often	12.5 (100)		13.3 (127)		13.0 (227)	
Checked your private messages without your permission (e.g. texts, WhatsApp, Facebook messenger)						
Never	70.0 (558)	1.9 (15)	67.1 (640)	1.4 (13)	68.4 (1198)	1.6 (28)
Once	8.7 (69)		12.1 (115)		10.5 (184)	
Few times	12.5 (100)		11.9 (114)		12.2 (214)	
Often	6.9 (55)		7.5 (72)		7.3 (127)	
Threatened to circulate or post sexual images or videos of you						
Never	93.4 (774)	1.9 (15)	89.6 (855)	1.5 (14)	91.3 (1599)	1.7 (29)
Once	2.8 (22)		4.9 (47)		3.9 (69)	
Few times	1.5 (12)		2.3 (22)		1.9 (34)	
Often	0.5 (4)		1.7 (16)		1.1 (20)	
Circulated or posted sexual images or videos of you						
Never	94.9 (756)	1.9 (15)	94.5 (902)	2.1 (20)	94.7 (1658)	2.0 (35)
Once	1.3 (10)		1.7 (16)		1.5 (26)	
Few times	1.0 (8)		1.0 (10)		1.0 (18)	
Often	1.0 (8)		0.6 (6)		0.8 (14)	
Shouted or screamed in your face, or called you hurtful names						
Never	64.1 (511)	2.1 (17)	62.9 (600)	1.5 (14)	63.4 (1111)	1.8 (31)
Once	14.7 (117)		13.0 (124)		13.8 (241)	
Few times	13.4 (107)		16.8 (160)		15.2 (267)	
Often	5.6 (45)		5.9 (56)		5.8 (101)	
Said negative things about your appearance or body						
Never	80.4 (641)	2.0 (16)	71.5 (682)	1.9 (18)	75.6 (1323)	1.9 (34)
Once	8.3 (66)		11.0 (105)		9.8 (171)	
Few times	6.4 (51)		11.7 (112)		9.3 (163)	
Often	2.9 (23)		3.9 (37)		3.4 (60)	
Threatened to hurt you physically						
Never	86.4 (689)	2.0 (16)	87.2 (832)	1.4 (13)	86.9 (1521)	1.7 (29)
Once	4.6 (37)		4.8 (46)		4.7 (83)	
Few times	4.1 (33)		3.6 (34)		3.8 (67)	
Often	2.8 (22)		3.0 (29)		2.9 (51)	
Punched, kicked, beaten you up or hit you with an object						
Never	86.3 (688)	2.0 (16)	88.8 (847)	1.5 (14)	87.7 (1535)	1.7 (30)
Once	5.3 (42)		4.3 (41)		4.7 (83)	
Few times	4.5 (36)		3.2 (31)		3.8 (67)	
Often	1.9 (15)		2.2 (21)		2.1 (36)	

BME = Black or minority ethnic group; GCSE = The General Certificate of Secondary Education.

### Measures

#### Socio-demographic characteristics

Participants self-reported their age, gender, sexual identity and ethnicity. Response options for gender were ‘male’, ‘female’ and ‘other’, with participants able to select multiple responses. Sexual identity was measured by asking participants which they currently most identify with: ‘Bisexual’, ‘Gay or lesbian’, ‘Heterosexual or straight’, ‘Rather not say’ and ‘Other’. Response options for ethnicity were: ‘White British’; ‘White not British’; ‘Mixed Race’; ‘Asian or Asian British’; ‘Black or Black British’; or ‘Other’.^37^ Responses were categorized as BME and other. Independent living was assessed by asking whether participants lived with a parent or other adult guardian. To measure spending money per week, participants were asked ‘How much money (in pounds) do you have to spend for yourself each week?’.^38^ Responses were categorized as ‘£20 or under’ and ‘over £20’ (i.e. having less than £3 a day was deemed to indicate little individual spending money in this age group). Educational attainment at age 16 was measured by asking whether participants had five or more GCSEs at A*–C. Responses were categorized as having five or more GCSEs at A*–C, or less than five GCSEs (including ‘no’ and ‘not sure’). Educational pathway was measured by asking respondents to indicate the qualification(s) currently studied in FE; categorized as those on an ‘academic educational pathway’ (AS/A-levels and Welsh Baccalaureate) versus a ‘non-academic educational pathway’ (all other courses).

#### Dating and relationships behaviours

##### Experience of dating and relationships

Participants were asked to report whether they had ever had a boyfriend or girlfriend, or been ‘seeing’ or ‘dating’ someone. Response options included ‘I am at the moment’, ‘I have in the past but not currently’ and ‘I have never had a boyfriend or girlfriend or been ‘seeing’ or ‘dating’ someone’. For the purpose of analysis, responses were categorized into ‘ever’ versus ‘never’.

##### Meeting partners online

Participants who reported ever having had a boyfriend or girlfriend were asked to report where they met their current or most recent boyfriend or girlfriend, or the person they had most recently been ‘seeing’ or ‘dating’. For the purpose of analysis, responses were categorized as ‘online’ and ‘other’.

##### Sending sexually explicit images

Participants who reported ever having had a boyfriend or girlfriend were asked whether they had ever sent someone a sexually explicit image of themselves (although not necessarily sent to their boyfriend/girlfriend). Response options included ‘No, never’, ‘Yes, once’ and ‘Yes more than once’. For the purpose of analysis, responses were categorized into ‘ever’ versus ‘never’.

##### Dating and relationship violence

Participants who reported dating or relationship experience were asked nine questions relating to whether they had experienced different types of DRV; controlling behaviours, verbal abuse, online sexual violence relating to sending sexually explicit images, and physical violence (see Online Resource 1). The questions, adapted from Barter *et al.*,^7^ were designed to be more inclusive of different types of DRV and to be age appropriate for 16–19 year olds. Response options were a four point Likert scale ‘Never’, ‘once’, ‘a few times’, ‘often’. Dating and relationship items were categorized and coded into binary variables to account for the severity and frequency of behaviours, with some questions not considered DRV if they had only occurred once (Online Resource 1).^7,9^

### Analysis

Exploratory factor analysis (EFA) was used to explore the underlying latent structure and relationships between the nine DRV items. EFAs were conducted separately for males and females due to the differing nature of DRV among genders.^9^ EFA with oblique rotation was conducted, extracting factors with eigenvalues >1 ,^39^ supported by the scree plot. Binary categories ‘Never experienced = 0’ versus ‘Ever experienced >1’ were then created for each of the DRV variables. These DRV variables were then used as outcomes in multilevel logistic regression models accounting for college-level clustering to examine the association between socio-demographic characteristics, sending explicit images, meeting partners online and different types of DRV victimization. Unadjusted models were estimated followed by adding all items into a fully adjusted model. Analysis was conducted in STATA 14.1.

## Results

Table 1 shows the experience of different types of DRV. Over 10% of students reported that they met their most recent partner online and 45.5% reported ever having sent a sexually explicit image. An EFA of the nine DRV items identified three factors for males explaining 50.3% of the variance; experience of threatening behaviours (34.1%, *α* = 0.768), online sexual violence (10.3%, *α* = 0.776) and controlling behaviours (5.9%, *α* = 0.620) and two factors for females, explaining 52.4% of the variance: threatening behaviours and online sexual violence (43.5%, *α* = 0.845) and controlling behaviours (8.9%, *α* = 0.701; see Online Resource 2).

Overall, 55.1% of males and 53.5% of females reported experiencing some form of DRV. Nearly half (49.9%) of males reported experience of controlling behaviours; 27.1% threatening behaviours, and 5.8% reported experience of online sexual violence. Similarly, nearly half of females’ experienced controlling behaviour (46.1%) and a third had experienced threatening behaviours (31.6%).

Table 2 presents the adjusted odds ratios at 95% confidence intervals for the association between DRV factors and socio-demographic characteristics, sexuality and dating behaviour (for unadjusted odds ratios see Online Resource 3). No consistent association was found between DRV and age, spending money per week, educational attainment or meeting partners online. The odds of experiencing online sexual violence were higher for younger, BME males and of experiencing threatening behaviour were higher for younger males. For females, the odds of experiencing controlling behaviour were lower for BME groups or those with less money to spend each week. The odds of experiencing any form of DRV were higher for females who lived independently and those reporting non-heterosexual identity. In the fully adjusted models, male students who had ever sent a sexually explicit image were more likely to report experiencing threatening behaviours (OR = 2.91, 95% CI: 2.01–4.23), online sexual violence (OR = 7.97, 95% CI: 3.63–17.52), and controlling behaviours (OR = 2.49, 95% CI: 2.05–3.02), than those who had not. Similarly, in fully adjusted models, female students who had sent a sexually explicit image were more likely to report experiencing online sexual violence (OR = 2.31, 95% CI: 2.04–2.62), and controlling behaviours (OR = 4.25, 95% CI: 3.43–5.26), than those who had not.

**Table 2:** Adjusted odds ratios (95% confidence intervals) for the association between DRV factors and socio-demographic characteristics, sexuality and dating behaviour

	Factor 1 (experience of threatening behaviours)		Factor 2 (experience of controlling behaviours)		Factor 3 (experience of online sexual violence)
	Males (*n* = 787)	Females (*n* = 933)	Males (*n* = 785)	Females (*n* = 947)	Males (*n* = 791)
Age					
18–19	1 (ref)	1 (ref)	1 (ref)	1 (ref)	1 (ref)
16–17	**0.81 (0.69**–0.94)*	0.87 (0.59–1.27)	0.98 (0.53–1.81)	1.02 (0.80–1.29)	**1.54 (1.01**–2.34)*
Spending money per week					
>£20	1 (ref)	1 (ref)	1 (ref)	1 (ref)	1 (ref)
<£20	0.80 (0.56–1.14)	1.05 (0.56–1.94)	0.81 (0.54–1.22)	**0.74 (0.60**–0.92)**	0.65 (0.21–2.08)
Ethnicity					
Non-BME	1 (ref)	1 (ref)	1 (ref)	1 (ref)	1 (ref)
BME	1.10 (0.49–2.43)	0.98 (0.72–1.33)	1.26 (0.56–2.81)	**0.66 (0.46**–0.95)*	**3.90 (1.72**–8.80)**
Educational pathway					
Academic pathway	1 (ref)	1 (ref)	1 (ref)	1 (ref)	1 (ref)
Non-academic pathway	1.12 (0.96–1.31)	0.96 (0.79–1.67)	1.47 (0.97–2.22)	0.92 (0.67–1.25)	1.16 (0.53–2.55)
Educational attainment					
>5 GCSEs	1 (ref)	1 (ref)	1 (ref)	1 (ref)	1 (ref)
<5 GCSEs	0.86 (0.58–1.28)	1.22 (0.78–1.89)	1.14 (0.83–1.55)	1.08 (0.72–1.63)	1.65 (0.96–2.84)
Living independently					
Not live independently	1 (ref)	1 (ref)	1 (ref)	1 (ref)	1 (ref)
Live independently	1.01 (0.40–2.55)	**4.03 (2.19**–7.41)***	0.73 (0.40–1.33)	***1.74 (1.33***–2.28)***	0.67 (0.12–3.66)
Sexual identity					
Heterosexual	1 (ref)	1 (ref)	1 (ref)	1 (ref)	1 (ref)
Other	1.88 (0.80–4.44)	**1.18 (1.01**–1.37)*	0.80 (0.45–1.42)	**2.09 (1.47**–2.96)***	1.78 (0.50–6.32)
Experience of meeting partners online					
No	1 (ref)	1 (ref)	1 (ref)	1 (ref)	1 (ref)
Yes	0.93 (0.55–1.59)	0.86 (0.65–1.15)	0.92 (0.68–1.25)	0.97 (0.53–1.77)	1.44 (0.77–2.65)
Ever sent sexually explicit image					
No	1 (ref)	1 (ref)	1 (ref)	1 (ref)	1 (ref)
Yes	***2.91 (2.01***–4.23)***	***2.31 (2.04***–2.62)***	***2.49 (2.05***–3.02)***	***4.25 (3.43***–5.26)***	***7.97 (3.63***–17.52)***

OR = odds ratio; AOR = adjusted odds ratio; Ref = reference. Statistically significant differences appear in bold italic text. *P < 0.05; **P < 0.01, ***P < 0.001. AOR: adjusted for age, spending money per week, ethnicity, educational pathway, educational attainment, living independently, sexual identity, experience of meeting partners online, ever sent sexually explicit image.

## Discussion

### Main finding of this study

In 16–19 year olds attending FE settings 55.1% of males and 53.5% of females reported experiencing some form of DRV. The most common form of DRV victimization was controlling behaviours, experienced by more than one-third of all young people with dating or relationship experience. Up to a third of males and females had experienced verbal DRV. The odds of experiencing a form of DRV were between 2–8 times greater for males and 2–4 greater for females who had ever sent a sexually explicit image of themselves, but DRV was not associated with meeting partners online.

### What is already known on this topic

The EFA of the nine DRV items identified a similar underlying structure to Breiding *et al.*’s^40^ typology of intimate partner violence. It separates physical, sexual and psychological or emotional violence. In line with previous literature, controlling behaviours were the most common form of DRV for both genders.^41^ Although an older sample was used in the current research, 31% of females in the current research reported their partner had told them who they could see or where they could go, compared to 30% reported by Barter *et al.*^7^ Contrary to existing literature which has identified gender differences in rates of DRV^5,6^ males’ DRV victimization was similar to females’. The high prevalence of DRV, especially controlling behaviours, may relate to the high prevalence of smartphone use among this population.^27^ This makes checking on a partner or their private messages easier than in the past. It could also reflect changing norms around sharing and monitoring public information online, the normalization of controlling behaviours or an increased willingness to report these behaviours.^42^

Around 45% of participants in this study reported sending sexually explicit images. This is similar to higher international (34%)^21^ and UK estimates (males, 32%; females, 44%).^5^ The association between sending sexually explicit images and DRV victimization supports previous literature where young people who reported DRV were at least twice as likely to have sent sexual content as those who had not experienced victimization.^5^ Coercion into sending/receiving such images may also reflect more nuanced controlling behaviour^43^ and so the association may reflect shared measurement variance. Images used as a form of currency can increase the risk of technology-assisted DRV including blackmail, revenge porn^43,44^ or wider cybervictimization.^45^

The odds of experiencing DRV were higher for females who lived independently. This may reflect increased relationship ‘seriousness’, associated with emotional investment and greater opportunity for conflict.^46^ Consistent with existing literature,^7,20^ DRV victimization was more common for females reporting non-heterosexual identity. Young people in same-sex relationships may experience unique homophobic stigma, violence and limited social support which may compound typical relationship difficulties, contributing to increased victimization.^7,20^

### What this study adds

The high prevalence of DRV coupled with a lack of differences across various socio-demographic characteristics suggests that DRV may be becoming normalized in 16–19 year olds and that universal intervention may be appropriate. Adolescence is a key period where norms are established and dating and relationship violence begins to manifest.^47^ The relationship between sharing sexually explicit images and DRV suggests that health education and promotion should be extended to include the potential for posting of these images without permission as a risk factor for DRV. The rapidly changing nature of young people’s social contexts, dating relationships, and increased Internet and social media use suggests more research is needed to contextualize the understanding of what young people think constitutes DRV and consider a behaviour which may increase their risk for experiencing DRV from the perspective of perpetrators and victims.

### Limitations of this study

Despite efforts to collect data in contrasting FE settings, the cross-sectional, non-random sample is not nationally representative (50.9% males, 49.1% females aged 16–19).^48^ Selection bias may be operating, such that students who had experienced DRV may have been more likely to respond; potentially resulting in higher DRV estimates. Similarly, as lifetime DRV was measured; those with multiple relationships would have higher opportunity to report multiple incidents. Rates of DRV were however similar to existing UK cross-sectional samples.^5^ Completion of the questionnaire required self-report data about dating behaviours. While every effort was made to ensure that participants completed questionnaires anonymously, individually and confidentially participants may have been unwilling to disclose DRV such that prevalence may be underestimated.^49^ Employing a family SES or neighbourhood deprivation measure may have yielded different results, and there is no established measure of individual SES in late adolescence. Variation in the definitions used to measure DRV provides additional challenges when collecting data and comparing research in this area. Sending sexually explicit images was also measured without relational context. As a cross-sectional study, associations may reflect reverse causality. For instance, sending explicit images may precede DRV.

## Supplementary Material

Supplementary Data

Supplementary Data

Supplementary Data

Supplementary Data

Supplementary Data
