# Supplementary material for: Dating and relationship violence among 16–19 year olds in England and Wales: a cross-sectional study of victimization
Source: J Public Health (Oxf). 2017 Nov 10;40(4):738–46. doi: 10.1093/pubmed/fdx139 (PMC6306090; doi:10.1093/pubmed/fdx139)
Supplement: Supplementary Data [file resubmissiontitlepage.docx]

**Dating and relationship violence among 16-19 year-olds in England and Wales: A cross-sectional study of victimisation**

***Dr Honor Young PhD^a^**

**Lecturer**

**^a^** DECIPHer, Cardiff University, UK, CF10 3BD, [youngh6@cardiff.ac.uk](mailto:youngh6@cardiff.ac.uk), +442922510085

**Ms Catherine Turney MSc^a^**

**PhD candidate**

**^a^** DECIPHer, Cardiff University, CF10 3BD, [TurneyC@cardiff.ac.uk](mailto:TurneyC@cardiff.ac.uk), +44292251008

**Dr James White PhD^b^**

**Senior Lecturer**

^b^ DECIPHer, Centre for Trials Research, Cardiff University, UK CF14 4YS, [WhiteJ11@cardiff.ac.uk](mailto:WhiteJ11@cardiff.ac.uk), +442920687054

**Professor Chris Bonell PhD^c^**

**Professor of Public Health Sociology**

^c^ Department of Social and Environmental Health Research, London School of Hygiene and Tropical Medicine, London, UK, WC1H 9SH, [Chris.Bonell@lshtm.ac.uk](mailto:Chris.Bonell@lshtm.ac.uk), +442076127918

**Dr Ruth Lewis PhD ^d, c^**

Visiting Assistant Professor

^d^ Department of Sociology, University of the Pacific, 3601 Pacific Avenue, Stockton, CA 95211, USA. [rlewis@pacific.edu](mailto:rlewis@pacific.edu), +1 209 9462895

^c^ Department of Social and Environmental Health Research, London School of Hygiene and Tropical Medicine, London, UK, WC1H 9SH.

**Professor Adam Fletcher PhD** **^e^**

Academic Director at Y Lab

**^e^** Y Lab, Cardiff University, UK, CF10 3AT, [FletcherA@cardiff.ac.uk](mailto:FletcherA@cardiff.ac.uk) +442920879874

**Corresponding author*

.
