# Supplementary material for: Dating and relationship violence among 16–19 year olds in England and Wales: a cross-sectional study of victimization
Source: J Public Health (Oxf). 2017 Nov 10;40(4):738–46. doi: 10.1093/pubmed/fdx139 (PMC6306090; doi:10.1093/pubmed/fdx139)
Supplement: Supplementary Data [file resubmissionacknowledgements.docx]

**Sources of Funding**

This work was supported by The Medical Research Council Public Intervention Development Scheme (MRC PHIND) grant number [MR/M026272/1].

**Acknowledgements**

The SaFE Project is a partnership between DECIPHer at Cardiff University, The London School of Hygiene and Tropical Medicine, The Institute of Education and the sexual health charity Brook, funded by the Medical Research Council Public Health Intervention Development Scheme (PHIND). The work was undertaken with the support of The Centre for the Development and Evaluation of Complex Interventions for Public Health Improvement (DECIPHer), a UKCRC Public Health Research Centre of Excellence. Joint funding (MR/KO232331/1) from the British Heart Foundation, Cancer Research UK, Economic and Social Research Council, Medical Research Council, the Welsh Government and the Wellcome Trust, under the auspices of the UK Clinical Research Collaboration, is gratefully acknowledged. We would like to thank the Further Education college staff and students for their time and contribution to the project, and members of our stakeholder advisory group for their guidance throughout the project. We would also like to thank Professor Chris Taylor for his help with the final manuscript.

**Conflicts of interest:** The authors declare that they have no conflict of interest.

**Clinical trials registry site and number:** Not applicable

**List of abbreviations**:

DRV – Dating and relationship violence

NVS - Non-volitional sex

STI – sexually transmitted infection

SES – socioeconomic status

BME - Black or minority ethnic group

GCSE - The General Certificate of Secondary Education

EFA - exploratory factor analysis

**Author Contributions:**

AF conceived the study, and participated in its design and coordination, interpretation of data and supported drafting the manuscript. HY participated in the study design, coordination, data analysis, interpretation of data and initial manuscript drafting. CB participated in the study design and coordination, interpretation of data and supported drafting the manuscript. RL participated in the study design and coordination, interpretation of data and supported drafting the manuscript. CT participated in the coordination of the study, collection of data and drafting of the manuscript; JW participated in the interpretation of the data and supported the drafting of the manuscript. All authors read and approved the final manuscript.
