# Supplementary material for: Dating and relationship violence among 16–19 year olds in England and Wales: a cross-sectional study of victimization
Source: J Public Health (Oxf). 2017 Nov 10;40(4):738–46. doi: 10.1093/pubmed/fdx139 (PMC6306090; doi:10.1093/pubmed/fdx139)
Supplement: Supplementary Data [file resubmissionauthoraffiliationsandresearchinterests.docx]

**Dating and relationship violence among 16-19 year-olds in England and Wales: A cross-sectional study of victimisation**

***Dr Honor Young PhD^a^**

**^a^** DECIPHer, Cardiff University, UK, CF10 3BD, [youngh6@cardiff.ac.uk](mailto:youngh6@cardiff.ac.uk), +442922510085

Dr Honor Young is a lecturer in quantitative methods at Cardiff University. Her research interests include sexual health and risk-taking behaviour, teenage pregnancy, dating and relationship violence, quantitative methods and youth participation in research design.

**Ms Catherine Turney MSc^a^**

**^a^** DECIPHer, Cardiff University, CF10 3BD, [TurneyC@cardiff.ac.uk](mailto:TurneyC@cardiff.ac.uk), +44292251008

Catherine Turney is a PhD candidate at Cardiff University. Her research interests include public health, sociological theory, visual sociology and educational transitions.

**Dr James White PhD^b^**

^b^ DECIPHer, Centre for Trials Research, Cardiff University, UK CF14 4YS, [WhiteJ11@cardiff.ac.uk](mailto:WhiteJ11@cardiff.ac.uk), +442920687054

Dr James White is a Senior Lecturer based at [DECIPHer](http://www.decipher.uk.net/) (Centre for the Development and Evaluation of Complex Public Health Interventions) a UK CRC Public Health Research Centre of Excellence, and the Deputy Director of Population Health Trials in the [Centre for Trials Research](http://www.cardiff.ac.uk/centre-for-trials-research). His research interests include how social, psychological, biological, behavioural and genetic factors from across the life course, influence behaviours which effect health and chronic diseases of major public health importance: cardiovascular disease and mental illness.

**Professor Chris Bonell PhD^c^**

^c^ Department of Social and Environmental Health Research, London School of Hygiene and Tropical Medicine, London, UK, WC1H 9SH, [Chris.Bonell@lshtm.ac.uk](mailto:Chris.Bonell@lshtm.ac.uk), +442076127918

### Professor Chris Bonell is a professor of public health sociology at the London School Hygiene and Tropical Medicine His research interests include adolescent health, sexual health, substance use and social exclusion and health, as well as in research methodology.

**Dr Ruth Lewis PhD ^d, c^**

^d^ Department of Sociology, University of the Pacific, 3601 Pacific Avenue, Stockton, CA 95211, USA. [rlewis@pacific.edu](mailto:rlewis@pacific.edu), +1 209 9462895

^c^ Department of Social and Environmental Health Research, London School of Hygiene and Tropical Medicine, London, UK, WC1H 9SH.

Dr Ruth Lewis is currently a visiting assistant professor at the University of the Pacific. Her research interests include Public Health, sex and sexuality, children and young people, families and qualitative methods.

**Professor Adam Fletcher PhD** **^e^**

**^e^** Y Lab, Cardiff University, UK, CF10 3AT, [FletcherA@cardiff.ac.uk](mailto:FletcherA@cardiff.ac.uk) +442920879874

Professor Adam Fletcher is a Professor in the School of Social Sciences and Academic Director of [Y Lab](http://ylab.wales/), the Public Services Innovation Lab for Wales. His research interests include public health improvement, public services innovation and how to increase the use of experimental designs in the social sciences.

**Corresponding author*
