# Supplementary material for: Dating and relationship violence among 16–19 year olds in England and Wales: a cross-sectional study of victimization
Source: J Public Health (Oxf). 2017 Nov 10;40(4):738–46. doi: 10.1093/pubmed/fdx139 (PMC6306090; doi:10.1093/pubmed/fdx139)
Supplement: Supplementary Data [file resubmission_onlineresource.docx]

**Online Resource 1: Coding of questions on Dating and Relationship Violence**

| **DRV items** | **Coding of DRV items** |
| --- | --- |
| Told them who they could see or where they could go? | ‘Never/Once’ = 0  ‘A few times’ = 1  ‘Often’ = 2 |
| Constantly checked up on what they were doing (e.g. by phone or texts)? |  |
| Checked their private messages without their permission (e.g. texts, WhatsApp, Facebook messenger)? |  |
| Shouted or screamed in their face, or called them hurtful names? |  |
| Said negative things about their appearance or body? |  |
| Threatened to circulate or post sexual images or videos of them? | ‘Never’ = 0  ‘Once’ = 1  ‘A few times’ = 2  ‘Often’ = 3 |
| Circulated or posted sexual images or videos of them? |  |
| Threatened to hurt them physically? |  |
| Punched, kicked, beaten them up or hit them with an object? |  |

DRV: Dating and Relationship Violence

**Online Resource 2: Factor loadings after rotation for nine individual dating and relationship items**

|  | Males (n=772) | | | | | | Females (n=920) | | |  |
| --- | --- | --- | --- | --- | --- | --- | --- | --- | --- | --- |
|  | F1  Threatening behaviour | F2  Online sexual violence | | F3  Controlling behaviour | | F1  Threatening behaviour and online sexual violence | | F2  Controlling behaviour | |  |
| Threatened to hurt them physically? | **0.844** | -0.030 | | -0.081 | | **0.794** | | 0.038 | |  |
| Punched, kicked, beaten them up or hit them with an object? | **0.698** | 0.015 | | -0.007 | | **0.784** | | 0.098 | |  |
| Shouted or screamed in their face, or called them hurtful names? | **0.595** | -0.057 | | 0.175 | | **0.403** | | 0.402 | |  |
| Said negative things about their appearance or body? | **0.425** | 0.201 | | 0.081 | | **0.537** | | 0.219 | |  |
| Threatened to circulate or post sexual images or videos of them? | -0.055 | **0.833** | | -0.020 | | **0.682** | | -0.027 | |  |
| Circulated or posted sexual images or videos of them? | 0.062 | **0.762** | | 0.036 | | **0.564** | | -0.090 | |  |
| Constantly checked up on what they were doing (e.g. by phone or texts)? | -0.066 | -0.083 | | **0.764** | | -0.092 | | **0.738** | |  |
| Checked their private messages without their permission (e.g. texts, WhatsApp, Facebook messenger)? | 0.064 | 0.065 | | **0.559** | | 0.126 | | **0.645** | |  |
| Told them who they could see or where they could go? | 0.110 | | 0.113 | | **0.520** | | 0.046 | | **0.790** | |

F= factor (based on eigenvalue>1 and scree plot)

**Online Resource 3. Unadjusted Odds ratios (95% confidence intervals) for the association between DRV factors and sociodemographic characteristics, sexuality and dating behaviour**

|  | **%** | **Factor 1 (Experience of threatening behaviours)** | | **Factor 2 (Experience of controlling behaviours)** | | **Factor 3 (Experience of online sexual violence)** | |
| --- | --- | --- | --- | --- | --- | --- | --- |
|  |  | **Males (n=787)** | **Females (n=933)** | **Males (n=785)** | **Females (n=947)** | **(Males n=791)** | |
| **Age** |  |  |  |  |  |  |  |
| 18-19 | 38.9 | 1 (ref) | 1 (ref) | 1 (ref) | 1 (ref) | 1 (ref) | |
| 16-17 | 61.1 | ***.72 (.56-.93)**** | .88 (.77-1.01) | .81 (.49-1.36) | .84 (.57-1.25) | 1.09 (.64-1.87) | |
| **Spending money per week** |  |  |  |  |  |  |  |
| >£20 | 66.0 | 1 (ref) | 1 (ref) | 1 (ref) | 1 (ref) | 1 (ref) | |
| <£20 | 29.6 | ***.71 (.52-.98)**** | ***.65 (.58-.72)****** | ***.70 (.52-.95)**** | .90 (.54-1.49) | .49 (.14-1.67) | |
| **Ethnicity** |  |  |  |  |  |  |  |
| Non-BME | 87.0 | 1 (ref) | 1 (ref) | 1 (ref) | 1 (ref) | 1 (ref) | |
| BME | 12.9 | 1.16 (.68-1.99) | *.62 (.47-.82)*** | 1.21 (.61-2.41) | .93 (.70-1.24) | ***2.56 (1.25-5.27)***** | |
| **Educational Pathway** |  |  |  |  |  |  |  |
| Academic pathway | 31.7 | 1 (ref) | 1 (ref) | 1 (ref) | 1 (ref) | 1 (ref) | |
| Non-academic pathway | 67.5 | 1.24 (.99-1.55) | .96 (.70-1.32) | ***1.55 (1.09-2.22)**** | 1.06 (.87-1.28) | 1.35 (.46-3.98) | |
| **Educational Attainment** |  |  |  |  |  |  |  |
| >5 GCSEs | 69.4 | 1 (ref) | 1 (ref) | 1 (ref) | 1 (ref) | 1 (ref) | |
| <5 GCSEs | 30.1 | .92 (.71-1.21) | .82 (.62-1.10) | 1.22 (.92-1.62) | 1.04 (.82-1.32) | 1.53 (.96-2.43) | |
| **Living independently** |  |  |  |  |  |  |  |
| Not live independently | 96.1 | 1 (ref) | 1 (ref) | 1 (ref) | 1 (ref) | 1 (ref) | |
| Live independently | 3.5 | 1.51 (.73-3.11) | **1.75 (1.46-2.09)***** | 1.01 (.45-2.29) | ***3.85 (2.45-6.06)****** | 1.25 (.41-3.77) | |
| **Sexual identity** |  |  |  |  |  |  |  |
| Heterosexual | 90.8 | 1 (ref) | 1 (ref) | 1 (ref) | 1 (ref) | 1 (ref) | |
| Other | 8.6 | ***2.25 (1.30-3.86)***** | **2.15 (1.71-2.71)***** | .96 (.67-1.40) | ***1.48 (1.22-1.81)****** | 2.57 (.86-7.73) | |
| **Experience of meeting partners online** |  |  |  |  |  |  |  |
| No | 86.6 | 1 (ref) | 1 (ref) | 1 (ref) | 1 (ref) | 1 (ref) | |
| Yes | 11.5 | 1.09 (.60-1.96) | 1.28 (.80-2.05) | .96 (.62-1.48) | 1.15 (.80-1.68) | 1.78 (.76-4.17) | |
| **Ever sent sexually explicit image** |  |  |  |  |  |  |  |
| No | 51.0 | 1 (ref) | 1 (ref) | 1 (ref) | 1 (ref) | 1 (ref) | |
| Yes | 44.5 | ***3.03 (2.37-3.87)****** | ***4.18 (3.42-5.09)****** | ***2.35 (1.80-3.08)****** | ***2.33 (2.02-2.70)****** | ***6.27 (3.29-11.96)****** | |

OR = Odds ratio; AOR = Adjusted odds ratio; Ref = reference. Statistically significant differences appear in ***bold italic*** text * p<.05; **p<.01, ***p<.001***.*** *AOR: adjusted for age, spending money per week, ethnicity, educational pathway, educational attainment, living independently, sexual identity, experience of meeting partners online, ever sent sexually explicit image.*
