# Supplementary material for: Dating and relationship violence among 16–19 year olds in England and Wales: a cross-sectional study of victimization
Source: J Public Health (Oxf). 2017 Nov 10;40(4):738–46. doi: 10.1093/pubmed/fdx139 (PMC6306090; doi:10.1093/pubmed/fdx139)
Supplement: Supplementary Data [file resubmissionresponsetoreviewers_jph-17-0386.docx]

**Response to reviewers comments for manuscript: JPH-17-0386**

11^th^ September 2017

Professor Ted Schrecker

Co-editor, Journal of Public Health

Dear Professor Schrecker,

**JPH-17-0386 - Dating and relationship violence and non-volitional sex among 16-19 year-olds in England and Wales: A cross-sectional study of victimisation. By Honor Young et al.**

Thank you for the reviewers’ comments on this manuscript and for the invitation to revise and resubmit. We are of course delighted to do so, and respond in a point-by-point manner below.

The structure of the discussion is currently as specified by the journal, and as reiterated by the editors. Two of the reviewers mention re-structuring the discussion to have a conclusive paragraph. We are happy to restructure the discussion in this way if the editors feel necessary.

We look forward to hearing from you.

Sincerely,

Honor Young

pp. co-authors

**Response to reviewers comments for manuscript: JPH-17-0386**

**Reviewer 1.**

Comments to the Author

An interesting paper, just a few suggestions below:

**1. There are two separate sets of key words, is this intentional?**

This is not intentional. The keywords defined by the authors “dating relationship violence, non-volitional sex, further education, colleges, adolescence” have been removed in favour of those defined by the journal. These have been clarified to include: “young people, educational settings and violence”

**2. Linked to this, I'm not convinced about the term 'non volitional' as a key word, I wouldn't search for this phrase.**

Non-volitional have been removed (as have the other keywords defined by the authors (dating relationship violence, non-volitional sex, further education, colleges, adolescence”). The keywords available as part of the journal have been selected “young people, educational settings and violence”. This now includes a keyword relating to violence.

**3. p3 - any more recent cost figure than this (2008)?**

These are the most recent UK figures for costs.

**4. p4 - should this say the age that 'most' young people leave education?**

The age at which young people in England formally leave education is 16 years. However recent legislation means that young people in England must do one of the following until they are 18; stay in full-time education (e.g., at college), start an apprenticeship or traineeship, or spend 20 hours or more a week working or volunteering, while in part-time education or training. https://www.gov.uk/know-when-you-can-leave-school . We acknowledge that not all young people will stay in education until aged 18 and therefore have included ‘most.’

**5. p5. you use the term UK when you mean England and Wales?**

We acknowledge that the data was collected in England and Wales and have therefore changed reference to the study sample, discussion and conclusions to reflect findings from England and Wales.

**6. do you use the term online dating synonymously with met a partner online? I think for this age group particularly they are not equitable. e.g. could meet on facebook, snapchat, forum etc. which is not what many people reading this paper will think of as 'online dating' (match.com, grindr, etc). Can you be clearer about this?**

We acknowledge this point. We have therefore changed reference to ‘online dating’ to refer to ‘meeting partners online.’ This has been changed throughout the manuscript and throughout the tables.

**7. suggest moving the what this study adds to be the last section rather than ending on limitations.**

As per the guidance from the editors, we have structured the discussion as follows:

Main finding of this study
What is already known on this topic?
What this study adds
Limitations of this study

If the editors feel this is suitable, we would be happy to move the ‘what this study adds’ section to after the limitations.

**Reviewer: 2**

Comments to the Author

I found this paper to be interesting and think it has the potential to help inform the field. That being said, I do have a few comments that I feel would improve the paper:

1.	**I would add the words “Teen Dating Violence” and “TDV” to your list of keywords as that is how DRV is most commonly referred to in the US. This will make your paper much easier to find for dating violence researchers.**

We have removed the additional keywords that were specified by the authors “dating relationship violence, non-volitional sex, further education, colleges and adolescence.” These have been removed in favour of those defined by the journal. These have been clarified to include: “young people, educational settings and violence.”

**2.	Page 6, Line 34 – You mention BME status. Please spell that out since it’s the first time you mention it.**

We have now included the definition “Black or Minority Ethnic group” for “BME”

**3.	Page 6, Line 38 – You mention GCSEs A*-C. Please spell that out since it’s the first time you mention it.**

We have now included the definition “General Certificates of Secondary Education” for “GCSE

**4.	Page 6, Line 55 – Were Asian and Asian British separated into two variables or were they combined? Same question for Black or Black British. If they were combined, why weren’t White British and White not British?**

Participants were asked to respond to whether they identify as ‘white British’ or ‘White not British’ or ‘Mixed Race’ or ‘Asian or Asian British’ or ‘Black or Black British’ or ‘Other’. This reflects the categorisation used in the Health Survey for England and Wales. Responses were categorised into Black Minority Ethnic group’ (BME) versus ‘other.’ This has been amended in the text to clarify.

**5.	Page 10, Line 23 – You have two periods after citation 5.**

The extra period has been removed.

**6.	My preference is not to end the paper with the limitations. I’ve found that it can leave a bad last impression for readers. I suggest putting a “Conclusions” section after the limitations.**

As per the guidance from the editors, we have structured the discussion as follows:

Main finding of this study
What is already known on this topic?
What this study adds
Limitations of this study

If the editors feel this is suitable, we would be happy to provide a conclusions section after the limitations.

7.	**Finally, I think that one of the strengths of your study was the fact that you asked the participants about their sexual orientation. However, I don’t feel that you elaborated on those findings enough. I would recommend saying more about the implications of those results.**

In the text we have written “Young people in same-sex relationships may experience unique homophobic stigma, violence and limited social support which may compound typical relationship difficulties, contributing to increased victimisation [7,20].” While we agree that one of the strengths of the research relates to asking about sexual identity, we are cautious to over-state this findings due to the small number of respondents who reported ‘non-heterosexual identity’ and the cross-sectional nature of the data.
